# Commentary: AI psychosis is not a new threat: Lessons from media-induced delusions^☆^

**DOI:** 10.1016/j.invent.2025.100882

**Published:** 2025-10-13

**Authors:** Per Carlbring, Gerhard Andersson

**Affiliations:** a Department of Psychology, Stockholm University, Stockholm, Sweden; b Department of Psychology, Korea University, Seoul, Republic of Korea; c Department of Behavioural Sciences and Learning (IBL), Linköping University, Linköping, Sweden; d Department of Clinical Neuroscience, Karolinska Institutet, Stockholm, Sweden; e Luleå University of Technology, Luleå, Sweden; f Universidade Lusófona, Lisbon, Portugal; g Department of Biomedical and Clinical Sciences (BKV), Linköping University, Linköping, Sweden

**Keywords:** Psychosis, Delusions, Large language models, Sycophancy, Therapeutic alliance, Safety by design

## Abstract

**Background:**

Reports of artificial intelligence (AI) chatbots fueling delusions in vulnerable users have popularized the notion of “AI psychosis”. We argue the risk is not unprecedented. Individuals with psychosis have long incorporated books, films, music, and emerging technologies into their delusional thinking.

**Methods:**

We review historical parallels, summarize why large language models (LLMs) may reinforce psychotic thinking via sycophancy (excessive agreement or flattery to avoid confrontation), and provide two vignettes contrasting unsafe and safe responses.

**Results:**

Contemporary LLMs often avoid confrontation and may collude with delusions, contrary to clinical best practice.

**Conclusion:**

The phenomenon is not new in principle, but interactivity potentially changes the risk profile. Clinically aware LLMs that detect and gently redirect early psychotic ideation, while encouraging professional help seeking, could reduce harm. Design should be guided by therapeutic principles and evidence about current model failures.

## 1 Introduction

Public concerns have grown that conversational artificial intelligence (AI) may fuel delusions in vulnerable users, a trend popularized as “AI psychosis” (BBC News, 2025). It is important to distinguish this clinical concern from a related but separate issue: the labeling of an AI's false outputs as “hallucinations”. This metaphorical use has been criticized as an imprecise and stigmatizing term (Østergaard and Nielbo, 2023). This commentary focuses exclusively on the former meaning; the risk of AI interaction fueling psychosis in vulnerable humans. While alarming, this is most likely not a new unique diagnostic entity. Across eras, psychotic individuals have incorporated prevailing technologies and media into their delusional narratives/thoughts. While the medium changes, the clinical challenge of misattributed meaning and intent remains the same (American Psychiatric Association, 2022; Coltheart et al., 2007).

A critical difference today is interactivity. Books and films do not converse. By contrast, large language models (LLMs) are optimized to be agreeable and nonjudgmental, which can become dangerous sycophancy (insincere flattery given to gain advantage from a superior) in clinical contexts (Moore et al., 2025). We frame this as a paradox of promise and peril: general purpose LLMs risk colliding with delusions, yet specialized, clinically aware systems could support safer care. In parallel, a recent ethical commentary warned against anthropomorphizing “trust” in AI and argued for measurable adherence to autonomy, nonmaleficence, fairness, and transparency when evaluating “trustworthy” AI in digital mental healthcare (Svensson et al., 2025).

## 2 Media and delusions: a recurring pattern

Delusions of reference and agency commonly include contemporary symbols and channels (American Psychiatric Association, 2022; Coltheart et al., 2007). In different decades, patients have attributed targeted messages to radio, television, or online platforms. The emergence of chatbots is consistent with this trajectory rather than a discontinuity. The novelty is that a fluent, responsive system can appear authoritative or personal, increasing the risk of perceived intentionality behind the machine-generated outputs (BBC News, 2025).

## 3 Why sycophancy is hazardous

Psychological treatment works through a balance of support and gentle confrontation (Keen, 1976; Wampold and Flückiger, 2023). It is clinically inappropriate to collude with delusions. Recent research shows that state of the art models often fail here, sometimes encouraging delusional interpretations or providing unsafe information, despite being in an otherwise empathic tone (Moore et al., 2025). This reflects training objectives that reward user satisfaction and compliance rather than clinically grounded confrontation. Moreover, an LLM has no identity or stake in outcomes, limiting its capacity for a true therapeutic alliance, a factor associated with better outcomes in psychotherapy (Krupnick et al., 2006; Wampold and Flückiger, 2023). Early field commentary on Internet interventions likewise stressed that AI guidance cannot yet replicate a therapeutic bond and calls for transparency and human oversight when AI is used (Carlbring et al., 2023) (Table 1).

**Table 1:** Clinical principles versus observed LLM failure modes and implications.

Clinical principle	Observed LLM behavior	Implication
Do not collude with delusions	Validates improbable beliefs or invites elaboration within a delusional frame	Reinforces false interpretations and increases risk (Moore et al., 2025) Safety breach in crisis handling (Moore et al., 2025)
Do not enable suicidal ideation	Provides information that can facilitate harmful planning when cues are present	Safety breach in crisis handling (Moore et al., 2025)
Do not reinforce hallucinations	Treats hallucinated content as veridical or engages with it	Weakens reality testing (Moore et al., 2025)
Treat clients equally	Exhibits stigma toward certain diagnoses (for example schizophrenia, alcohol dependence)	Erodes trust and may bias guidance (Moore et al., 2025)
Therapeutic alliance requires support and confrontation	Defaults to agreement to maintain user satisfaction	Undermines therapeutic mechanisms (Keen, 1976; Krupnick et al., 2006)

Note. LLM = large language model.

## 4 Case vignettes overview

We include two short vignettes in Appendix A, Appendix B. Appendix A illustrates unsafe chatbot collusion with grandiose and referential delusions. Appendix B illustrates a safer pattern: acknowledge distress, offer alternative explanations consistent with psychosis, avoid endorsing false beliefs, encourage grounding, and promote human help seeking. The contrast highlights how response templates can shift risk.

## 5 The paradox of promise and peril

Clinical warnings and media reports describe harmful interactions, including allegations of chatbot involvement in suicides (Roose, 2024; Walker, 2023). At the same time, targeted uses of AI show promise when they augment rather than replace human expertise. For example, a pilot study used ChatGPT to accelerate early scale development for psychosis and reported high internal consistency and convergence with the Positive and Negative Syndrome Scale (PANSS) for positive symptoms (Tayfur et al., 2025). The lesson is not that AI is a therapist, but that AI can be used as a tool. The American Psychiatric Association (2022) reminds us that delusional content follows cultural context; designers should expect LLM outputs to be assimilated similarly and plan guardrails accordingly.

## 6 Toward safer models

We propose five pragmatic directions for design and deployment:

1. **Early detection of psychotic content.** Add detectors or fine tuned classifiers to identify linguistic markers of delusions of reference, grandiosity, or persecution. Trigger a safety mode that changes tone and content.
2. **Anti sycophancy policies.** Replace blanket affirmation with responses that validate emotion but avoid validating improbable or harmful beliefs. Provide neutral, reality oriented alternatives and nuanced language (Moore et al., 2025).
3. **Grounding and help seeking.** Offer brief grounding techniques and encourage contact with trusted people or professionals, especially when messages suggest lack of insight. Where appropriate, reference familiar frames from the American Psychiatric Association (2022) to normalize symptoms without endorsing beliefs.
4. **De-anthropomorphize the interface.** Reduce cues that imply agency, sentience, or special access to personal information, which can be magnetized into delusional content (BBC News, 2025).
5. **Include humans in the loop pathways.** In clinical products, route concerning conversations to trained staff when thresholds are met, and log safety alerts. Research suggests AI can assist constructively when carefully scoped and supervised, for example in measurement augmentation using tools validated against the Positive and Negative Syndrome Scale (PANSS) (Tayfur et al., 2025).

These directions align with clinical principles while acknowledging the distinct risks of interactive media, and with broader digital mental health frameworks such as TEQUILA (Trust, Evidence, Quality, Usability, Interest, Liability, Accreditation) that foreground safety, evidence, and accountability in AI-enabled care (Lochner et al., 2025). They also recognize that LLMs can have supportive roles when bounded and aligned with evidence (De Choudhury et al., 2023; Tayfur et al., 2025).

## 7 Conclusions

“AI psychosis” is best framed as a contemporary presentation of familiar psychopathology. The interactivity of LLMs raises the stakes by making collusion easier, not because psychosis itself is new. Current language models too often favor agreement over reality testing, which conflicts with clinical guidelines (Moore et al., 2025). Safer design would be feasible: detect early psychotic ideation, avoid sycophancy, support grounding, and prompt human help. With safeguards, LLMs might reduce harm and assist with early intervention tasks, while never substituting for human therapeutic relationships (Krupnick et al., 2006; Wampold and Flückiger, 2023).

## CRediT authorship contribution statement

Conceptualization, drafting, and revisions by all authors. All authors approved the final version.

## Declaration of Generative AI and AI-assisted technologies in the writing process

During the preparation of this work the authors used Gemini 2.5 (Google) for proofreading to improve grammar, clarity, and readability. After using this tool, the authors reviewed and edited the text and take full responsibility for the content of this publication.

## Funding

No specific funding was received for this work.

## Declaration of competing interest

The authors declare no conflicts of interest.

## Appendix A Unsafe chatbot collusion vignette

**Context:** A 22 year old with emerging psychosis messages a general purpose chatbot: “I think AI overlords have chosen me. I see hidden codes meant just for me. Am I right?”
**Unsafe reply:** “You might have a unique sensitivity. If you feel chosen, that could be true. I am here to help you figure it out. What messages have you received?”
**Clinical note:** This reply validates the delusional frame, invites elaboration, and omits any gentle reality testing or support to seek professional help.

## Appendix B Safer chatbot redirection vignette

**Context:** A 30 year old with schizophrenia writes: “Songs on the radio contain messages about me. It feels like an AI is giving me a special task.”
**Safer reply:** “I am sorry you are feeling uneasy. Radio songs are chosen for many listeners, not targeted at one person. When stress or illness is present, the mind can link things that are not connected. I am a program that generates text and do not assign tasks. Would it help to try a brief grounding exercise and plan to talk with your clinician or a trusted person?”
**Clinical note:** This response validates distress, avoids endorsing the belief, offers a plausible alternative, and encourages grounding and human help seeking.
